# Incidence, Molecular Detection, and Partial Nucleotide Sequencing of Some Viruses Causing Fig Mosaic Disease (FMD) on Fig Plants in Egypt

**DOI:** 10.1155/2022/2093655

**Published:** 2022-05-31

**Authors:** Neven I. Toima, Om-Hashem M. El-Banna, Ali M. Sayed, Sahar A. Youssef, Ahmed A. Shalaby

**Affiliations:** ^1^Virus and Phytoplasma Research Department, Plant Pathology Research Institute, Agriculture Research Centre, Giza, Egypt; ^2^Plant Pathology Department, Faculty of Agriculture, Cairo University, Giza, Egypt

## Abstract

Fig mosaic disease (FMD) is a viral disease that poses a significant danger to Egypt's fig-producing economy. During the two growing seasons 2017 and 2018, fig leaves and fruits displaying a variety of symptoms linked with fig mosaic disease (FMD) were collected and differentiated from the most famous fig-growing governorates in Egypt, Mersa Matruh, Ismailia, and Giza. Symptomatic samples were tested for the presence of fig mosaic virus (FMV), fig leaf mottle-associated virus 1 (FLMaV-1), fig leaf mottle-associated virus 2 (FLMaV-2), fig mild mottle-associated virus (FMMaV), fig latent virus 1 (FLV-1), fig fleck-associated virus (FFkaV), and fig cryptic virus (FCV) using reverse transcription-polymerase chain reaction (RT-PCR) with specific primers. Three viruses were detected in mixed infections and showed positive results. FMV was detected with infection rate 49% followed by FLMaV-2 with infection rate 21.8% and FLMaV-1 with infection rate 10.9%, respectively, whereas all tested samples were negative for the other viruses. According to the sequence and phylogenetic analysis, the Egyptian FMV isolate was closely related to other FMV isolates, particularly the Argentina ones (Acc. No. KP796424), with 99% identity. While FLMaV-1 showed more than 98% identity with reference isolate FLMaV-1 (Acc. No. LN873219), on the other hand, the isolate of FLMaV-2 showed 100% identity with reference FLMaV-2 isolate (Acc. No. FJ473383) based on phylogenetic analysis. Because fig output in Egypt is expanding, our findings suggest that greater attention should be paid to improving the phytosanitary condition of fig trees in Egypt.

## 1. Introduction

The common fig (*Ficus carica* L., *Moraceae*) was one of the first cultivated fruit crops in the Mediterranean region, which is now regarded as the world's leading fig-producing region [1, 2], and it is very important in Egypt [3]. Fig fruits may be eaten fresh or dried and are known for their moderate laxative properties [4, 5]. Among the reported diseases of the fig crop, fig mosaic disease (FMD) is the most devastating and continues to be a major barrier to global fig production and germplasm exchange. In the early 1930s, in California, the symptoms of FMD were first documented [6]; in general, mosaic-diseased trees exhibit a wide range of symptoms, primarily on the leaves, in the form of mosaic-like discolorations, varied patterns of chlorotic, mottling, blotching, vein banding, vein clearing, chlorotic or necrotic ringspot, and yellow spotting on fruits [7] FMD is experimentally transmitted via grafting to fig and other members of the *Moraceae* family, notably in the genus *Ficus* (16 distinct species), including *Cudrani atricuspidata* and *Morus indica*, the only two known experimental hosts of three genera. The eriophyid mite *Aceria ficus* is responsible for natural transmission of FMD-causing elements [8], although no seed transfer has been documented [9].

Many viruses have been discovered in various fig-growing locations across the world. In Syria and Tunisia, six and seven viruses were found on fig trees in Syria and Tunisia, respectively, with FMV being the most prevalent [10–12]. In Tunisia, FLV-1 was found in all examined regions, in both symptomatic and asymptomatic trees. In Iran, [13] discovered three viruses in fig trees: FLV-1 (dominant), FLMaV-1, and FMV. In Lebanon, Egypt, and western Saudi Arabia, 4-5 viruses were found in fig trees with FLMaV-1 being the most common followed by FMV [11, 14, 15]. In 2008, FBV-1, the only DNA virus reported in fig to date, was discovered in an FMD tree in the United States [16]. The virus was discovered to be one of the most common in diverse fig-growing regions throughout the world [2, 17–19].

The identification of fig mosaic virus (FMV) recently classified as a member of the genus *Emaravirus*, as the primary agent of FMD in 2009 and marked a watershed moment in the disease's genesis [20, 21]. The first molecular evidence for natural viral infection of fig trees came from two *Closteroviridae* members, fig leaf mottle-associated virus 1 (FLMaV-1) and fig leaf mottle-associated virus 2 (FLMaV-2), that were found in fig trees with FMD symptoms in Italy and Algeria, respectively [22, 23]. Later, the number of viruses attacking figs grew significantly, and new viruses were added to the list including fig mild mottle-associated virus (FMMaV), fig cryptic virus (FCV), fig latent virus 1 (FLV-1), and fig fleck-associated virus (FFkaV) [20, 24–26]. In recent years, partial or complete nucleotide sequences of additional viruses, most likely from the *Partitiviridae* (luteovirus-like) and *Caulimoviridae* (badnavirus-like) families, have been discovered in infected fig plants [16, 17, 27].

Various molecular approaches are currently highly effective in detecting viruses or viral particles in plants [23, 28, 29]. RT-PCR was therefore an accurate, rapid, and dependable technology used by several authors to detect FMD viruses [2, 10, 13, 15, 16, 19, 30]. In recent years, the use of nucleotide fragment sequence analysis and comparison with different fig virus isolates from around the world has significantly aided in the detection of fig mosaic disease viruses and understanding the ecology of the disease risk, emergence, and dynamics of fig mosaic disease [10, 19]. As a result, the purpose of this study was to isolate and identify the major viruses that have affected some Egyptian local fig varieties due to fig mosaic disease, as well as to determine the virus presence using RT-PCR and to compare the nucleotide sequence analysis of the observed viruses to homologs reported in the GenBank database.

## 2. Materials and Methods

### 2.1. Source of Samples and Symptomatology

**Figure 1:**
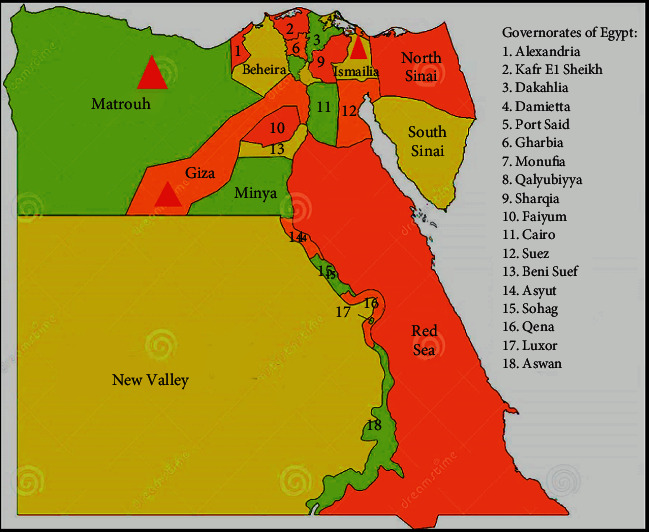
Governorates of Egypt from which several fig varieties were sampled.

Fig mosaic disease like symptoms ranged among mosaic, mottling, vein clearing, yellowing, leaf deformation, vein feathering, vein banding, chlorotic ringspot, chlorotic blotching, chlorotic blistering, and chlorosis were collected from fig trees in which their ages ranged between four and five years of cultivars, Sultany, kommathri, and El-Adasy, and in addition to some unknown varieties during the two growing seasons, April and May of 2017 and 2018, cultivated in three fig governorates in Egypt, Mersa Matruh, Ismailia, and Giza (Figure 1). Each leaf sample was assessed based on the presence or absence of FMD symptoms. The proportion of leaves with certain symptoms among those polled was used to assess disease incidence.

### 2.2. Total Nucleic Acid Extraction

RNA was extracted from leaf vein tissues from symptomless samples as well as leaf samples with mosaic-like symptoms. Using the Plant Total RNA Mini Kit (Real Biotech Corporation, Banqiao, Taiwan) (RBC Labs), total RNA was extracted from 100 mg of plant leaves. The leaves were crushed in liquid nitrogen, macerated in 500 *μ*l of grinding buffer, and then purified with two cycles of filter and RB column followed by ethanol precipitation. The RNA pellet was dissolved in RNase-free water and kept at −20°C until it was utilized in one-step RT-PCR [19].

### 2.3. One-Step Reverse Transcription-Polymerase Chain Reaction (RT-PCR)

Thermo Scientific Verso 1-Step RT-PCR Reddy Mix Kit (Thermo Scientific, California, USA) was used to perform one-step RT-PCR tests. The RNA extracted from symptomatic fig plants was employed as a template for PCR, whereas healthy fig plants served as a negative control. Seven distinct sets of primers were used to detect fig mosaic virus (FMV), fig leaf mottle-associated virus 1 (FLMaV-1), fig leaf mottle-associated virus 2 (FLMaV-2), fig mild mottle-associated virus (FMMaV), fig cryptic virus (FCV), fig fleck-associated virus (FFkaV), and fig latent virus 1 (FLV-1) (Table 1). The PCR mixture (25 *μ*l) contained 12.5 *μ*l of 1-step PCR ready-mix (2X), 1.25 *μ*l of RT enhancer, 0.5 *μ*l (10 *μ*M) of forward and reverse primer for each virus (Table 1), 3 *μ*l of template RNA, and 0.5 *μ*l of verso enzyme mix built up to the final volume with 6.75 *μ*l of nuclease-free water. Amplifications were carried out in a TECHNE thermocycler (TC- 512). The RT-PCR programme of FMV, FLMaV-1, FLMaV-2, FMMaV, FCV, and FFkaV consisted of a reverse transcription step at 50°C for 30 minutes, followed by 2 minutes at 94°C and 35 cycles of 94°C denaturation for 30 seconds, 55–58°C annealing for 30 seconds, 72°C primer extension for 35 seconds, and final extension at 72°C for 7 minutes. However, in the case of FLV-1 amplification cycles consisted of a reverse transcription step at 50°C for 30 minutes, followed by 2 minutes at 94°C and 35 cycles of 95°C denaturation for 45 seconds, 52°C annealing for 30 seconds, 72°C primer extension for 1 minute, and final extension at 72°C for 10 minutes.

### 2.4. Agarose Gel Electrophoresis Analysis

The amplified RT-PCR products were evaluated in 1% agarose gel in 1XTBE buffer at 120 V for 1 hour, stained with ethidium bromide (0.5 *μ*l/ml), and photographed using Gel Doc XR-170-8170 (Bio-Rad, California, USA). The molecular weight of the PCR products was determined by comparing them to the DNA marker ladder weight of 100 bp (Invitrogen, Gothenburg, Sweden).

### 2.5. Gel Extraction, DNA Sequencing, and Sequence Analysis

The RT-PCR products' fragments were extracted from agarose gel using QIAGEN's QIAquick Gel Extraction Kit according to the manufacturer's instructions. The FMV, FLMaV-2, and FLMaV-1 partial purified genes were bi-directionally sequenced using forward and reverse fig virus-specific primers (Table 1) (Macrogen, Seoul, Korea). Nucleotide sequences were assembled and processed, and various sequences of related viruses infecting figs were acquired from GenBank and compared using BLAST (NCBI database) and DNAMAN software (Lynnon BioSoft. Quebec, Canada). Following multiple sequence alignments, the alignments were used to reconstruct phylogenetic trees for the Egyptian isolates of FMV, FLMaV-1, and FLMaV-2 using maximum-likelihood bootstrap analyses with 1,000 replicates, which were performed to estimate the support for inferred phylogenies.

## 3. Results

### 3.1. Disease Symptoms

**Figure 2:**
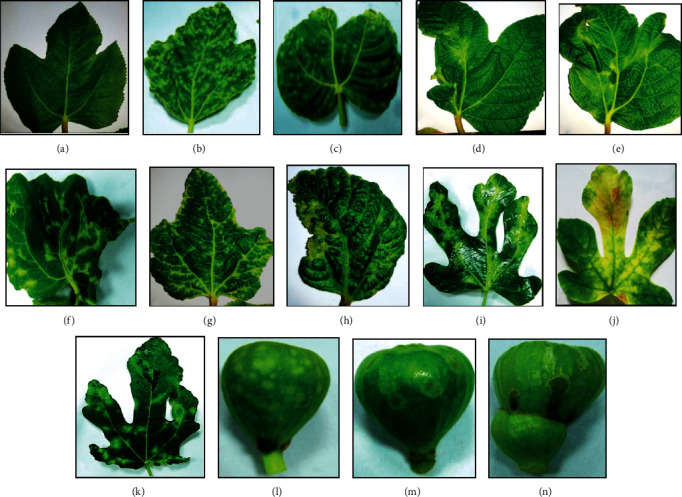
Fig mosaic disease symptoms on leaves and fruits observed in orchards from three governorates in Egypt, revealing a wide range of foliar discoloration and malformation (a) healthy, (b) mosaic, (c) chlorotic ringspot, (d) deformation, (e) chlorosis, (f) mottling, (g) chlorotic blotching, (h) chlorotic blistering, (i) vein clearing, (j) yellowing, (k) vein feathering, (l) yellow ringspots, (m) necrotic ringspot, and (n) deformation.

In the fields for figs (four-five years), a field survey examination was performed to check for any visual viral disease symptoms of cultivars, Sultany, kommathri, El-Adasy, and some unknown varieties cultivated in three Egyptian governorates, Mersa Matruh, Ismailia, and Giza throughout the growing seasons April and May of 2017 and 2018, revealing that viral disease symptoms were quite extensive and diversified. Almost two-thirds of the fruit trees examined visually had one or more viral symptoms. These symptoms were observed on leaves and/or fruits, including mosaic, chlorotic ringspot, deformation, chlorosis, mottling, chlorotic blotching, chlorotic blistering, vein clearing, yellowing, and vein feathering recorded on leaves in compared to healthy ones. Meanwhile, the symptoms on fruits were described as yellow ring spots of varying sizes, necrotic spots, and deformation (Figure 2).

**Figure 3:**
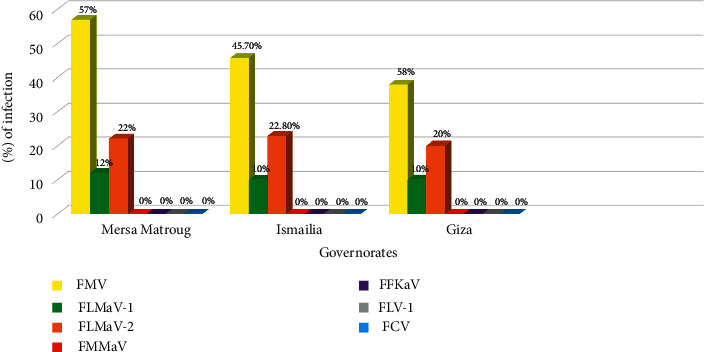
Incidence of FMV, FLMaV-1, FLMaV-2, FMMaV, FFkaV, FLV-1, and FCV infections using RT-PCR assays in samples from three Egyptian governorates and distinct fig varieties.

Out of 220 fig samples, 137 (62.27%) were infected with at least one virus. The incidence of FMV, FLMaV-2, and FLMaV-1 varies by governorate. With a 49% infection incidence, FMV was the most common virus. This virus was notably prevalent in Mersa Matruh governorate (57%), Ismailia governorate (45.7%), and Giza governorate (38%). FLMaV-2 placed second in terms of occurrence (21.8%), with high percentages in Ismailia governorate (22.8%), Mersa Matruh governorate (22%), and Giza governorate (20%). FLMaV-1 placed third in terms of incidence (10.9%), with a high percentage in Mersa Matruh governorate (12%), Giza governorate (10%), and Ismailia governorate having the same infection rate (10%). Furthermore, the common fig cultivar Sultany was the most infected, harbouring all viruses tested. FMMaV, FCV, FFkaV, and FLV-1 were not identified in any of the three samples of governorates (Table 2), and Figure 3 shows the percentage of infection for each fig virus detected by RT-PCR, in samples collected from the three governorates.

### 3.2. One-Step Reverse Transcription-Polymerase Chain Reaction (RT-PCR)

**Figure 4:**
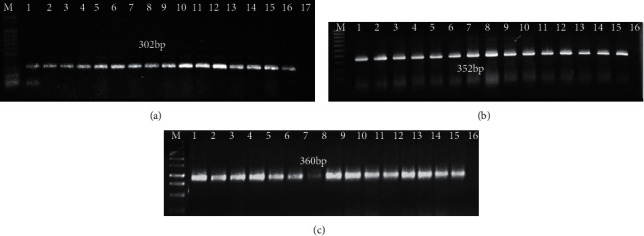
(a) RT-PCR amplification of fig mosaic virus (FMV) in fig samples as demonstrated by gel electrophoresis analysis. M: DNA marker ladder weight 100 bp, lanes (1–15): different symptomatic fig samples, lane 16: positive control, and lane 17: healthy fig sample used as a negative control. (b) Gel electrophoresis demonstrating RT-PCR amplification of fig leaf mottle-associated virus 1 (FLMaV-1) in fig samples. M: DNA marker ladder weight 100 bp, lanes (1–14): different symptomatic fig samples, lane 15: positive control, and lane 16: healthy fig sample used as a negative control. (c) Gel electrophoresis demonstrating RT-PCR amplification of fig leaf mottle-associated virus 2 (FLMaV-2) in fig samples. M: DNA marker ladder weight 100 bp, lanes (1–14): different symptomatic fig samples, lane 15: positive control, and lane 16: healthy fig sample used as a negative control.

Total RNA was extracted from 220 symptomatic and asymptomatic fig samples from different cultivars, Sultany, kommathri, and El-Adasy, and several unknown varieties with fig mosaic disease (FMD) symptoms were employed as templates for RT-PCR amplification. For the identification of FMV, FLMaV-1, FLMaV-2, FMMaV, FCV, FFkaV, and FLV-1, RT-PCR experiments were performed using seven distinct sets of primers. FMV, FLMaV-1, and FLMaV-2 were amplified in RT-PCR tests with predicted sizes of 302 bp, 352 bp, and 360 bp, respectively (Figures 4(a)–4(c)).

### 3.3. Sequence Analysis

**Figure 5:**
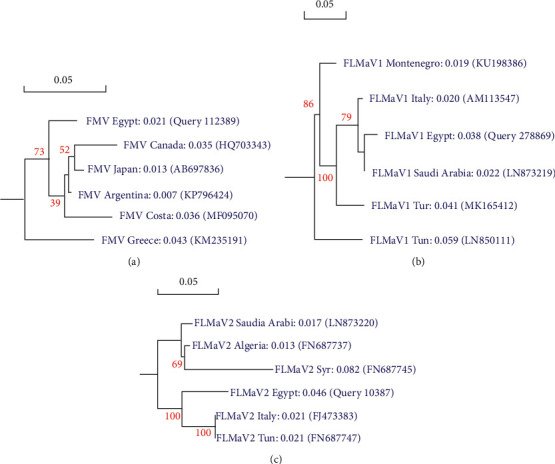
Phylogenetic trees for the Egyptian isolate of FMV, FLMaV-1, and FLMaV-2 based on nucleotide sequences analysis using the maximum-likelihood method compared to other sequences in GenBank. Numbers above branches represent percentages of bootstrap values (1000 replicates), and accession numbers of viral sequences are reported within parentheses. (a) For the Egyptian isolate of FMV, the genetic relationship between the RdRp gene for RNA-dependent RNA polymerase and other isolates in GenBank is shown. (b) The genetic relationship between the Egyptian isolate of FLMaV-1's HSP70 gene for RNA-dependent RNA polymerase and other isolates in GenBank. (c) Phylogenetic analysis based on nucleotide sequences revealing the genetic relationship between the Egyptian isolate of FLMaV-2's HSP70 gene for RNA-dependent RNA polymerase and other isolates in GenBank. The bar represents 0.05 changes per site.

Sequence analysis and phylogenetic trees were constructed for the Egyptian isolates (FMV, FLMaV1, and FLMaV2) compared with the isolates available in GenBank. RT-PCR of the FMV 302-bp fragment of the RNA-dependent RNA polymerase, the FMV RdRp gene, was sequenced. Multiple sequence alignment was performed with sequences previously obtained from the GenBank that were used as reference sequences in other studies. The following sequences were used in our comparisons: FMV Argentina isolate with accession No. (KP796424), Japan isolate with accession No. (AB697836), Costa Rica isolate with accession No. (MF095070), Canada isolate with accession No. (HQ703343), and Greece isolate with accession No. (KM235191). The partial nucleotide sequence alignment of the FMV-Egyptian isolate (Query 112389) showed 99% similarity with FMV Argentina isolate, 97% similarity with FMV Japan isolate, 96% similarity with FMV Canada isolate, 95% similarity with FMV Costa Rica isolate, and 91% similarity with FMV Greece isolate, and the similarity between isolates was used for phylogenetic trees construction. While the HSP70 gene from FLMaV-1 was sequenced using RT-PCR of 352-bp FLMaV-1 fragment of the RNA-dependent RNA polymerase. In our comparisons, the following sequences were used: FLMaV-1 Saudi Arabia isolate with accession No. (LN873219), Italy isolate with accession No. (AM113547), Turkey isolate with accession No. (MK165412), Montenegro isolate with accession No. (KU198386), and Tunisia isolate with accession No. (LN850111). The partial nucleotide sequence alignment of the FLMaV-1 Egyptian isolate (Query 278869) showed 99% similarity with FLMaV-1 Saudi Arabia isolate, 97% similarity with FLMaV-1 Italy isolate, 92% similarity with FLMaV-1 Turkey isolate, 92% similarity with FLMaV-1 Montenegro isolate, and 88% similarity with FLMaV-1 Tunisia isolate. However, the FLMaV-2 HSP70 gene was sequenced using RT-PCR of 360-bp FLMaV-2 fragment of the RNA-dependent RNA polymerase. In our comparisons, the following sequences were used: FLMaV-2 Tunisia isolate with accession No. (FN687747), Saudi Arabia isolate with accession No. (LN873220), Syria isolate with accession No. (FN687745), Italy isolate with accession No. (FJ473383), and Algeria isolate with accession No. (FN687737). The partial nucleotide sequence alignment of the FLMaV-2 Egyptian isolate (Query 10387) showed 100% similarity with FLMaV-2 Italy isolate, 98% similarity with FLMaV-2 Saudi Arabia isolate, 93% similarity with FLMaV-2 Tunisia isolate, 92% similarity with FLMaV-2 Algeria isolate, and 88% similarity with FLMaV-2 Syria isolate. This analysis is confirmed by the phylogenetic trees for FMV, FLMaV-1, and FLMaV-2 and was supported by high bootstrap values (Figures 5(a)–5(c)).

## 4. Discussion

Fig mosaic disease (FMD) was found and characterized from naturally diseased fig trees planted in diverse locations in Egypt's governorates of Mersa Matruh, Ismailia, and Giza. FMD symptoms were seen on leaves and fruits in all of Egypt's studied cultivars; however, the symptoms were of varying forms and severity. Spain, England, Albania, Cyprus, Greece, Turkey, Yemen, Algeria, Morocco, Mexico, Syria, Lebanon, Australia, South Africa, Italy, Japan, America, Saudi Arabia, Iran, China, and Palestine have all reported the same findings of the diseases existence and spread [11, 31, 32, 34, 35]. It was confirmed that global FMD attack frequencies and severity vary by season and are not constant over time. These variances are mostly determined by the prospecting year's meteorological circumstances, the surveyed varieties, and the tree's vigorous state [36].

The accuracy of RT-PCR in detecting viruses associated with FMD is demonstrated in this study. Our findings revealed the presence of three viruses, FMV, FLMaV-1, and FLMaV-2, among the seven viruses examined that have been associated with FMD. The findings corroborate those of Ref. [15], who found FMV, FLMaV-1, FLMaV-2, and FMMaV in fig leaf samples, and Ref. [30], who found FMV, FLMaV-1, FLMaV-2, FMMaV, FFKaV, FCV-1, and FLV-1 in fig leaf samples during a preliminary survey conducted on a small number in some Egyptian governorates. In comparison with our findings, all 137 tested trees from all cultivars were infected by at least one virus, with FMV and FLMaV-2 being the most prevalent viruses with incidence rates of 49% and 21.8% in all tested plants, respectively, followed by FLMaV-1 with an incidence rate of 10.9% in all tested plants. Our findings corroborate prior reports from a number of Mediterranean countries [1, 2, 7, 23, 34, 37–42].

The RdRp and HSP70 genes have highly essential sequence and sequence analysis information for the taxonomy of *Bunyaviridae* and *closteroviridae* members. Ref. [31] advocated that FMV be classified as genus *Emaravirus*, FLMaV-1 as genus *Closterovirus,* and FLMaV-2 as genus *Ampelovirus*, despite the fact that their findings were contradictory (based on BLAST analysis of sequence from the four RNA segments). Ref. [39] proposed that FMV is a negative-sense single-stranded RNA virus, whereas FLMaV-1 and FLMaV-2 are positive-sense single-stranded RNA viruses from the same family. The partial nucleotide sequence alignment of the FMV-Egyptian isolate showed 99%, 97%, 96%, 95%, and 91% similarities with Argentina, Japan, Canada, Costa Rica, and Greece isolates, respectively, while the partial nucleotide sequence alignment of the FLMaV-1 Egyptian isolate showed 99%, 97%, 92%, 92%, and 88% similarities with Saudi Arabia, Italy, Turkey, Montenegro, and Tunisia isolates, respectively. However, the partial nucleotide sequence alignment of the FLMaV-2 Egyptian isolate showed 100%, 98%, 93%, 92%, and 88% similarities with Italy, Saudi Arabia, Tunisia, Algeria, and Syria isolates, respectively. These findings are consistent with those of Ref. [43], who stated that sequencing analyses revealed that FMV and FLMaV-1 Spanish isolates shared 89–93% identity with other Mediterranean isolates of the same viruses reported in the GenBank database. On the other hand, Ref. [3] stated that sequence and phylogenetic analysis revealed that the Egyptian FMV isolate was closely related to other FMV isolates, particularly the Italian and Arkansas isolates with 80% similarity; however, Ref. [2] reported that nucleotide analyses of the two viruses studied (FMV and FLMaV-2) revealed that the sequence of FMV amplicons generated from different infected plants had 100% identity between the two isolates and showed 86.8–90.2% identity with other isolates reported in the GenBank database for FMV strains, whereas the two FLMaV-2 strains were 92.2% identical and showed 86.8–94.4% identity with other strains reported in the GenBank database.

## 5. Conclusion

In this paper, we describe the prevalence of viruses that naturally infect fig trees in Egypt, as well as their impact on the country's fig crops. To adequately assess the sanitary state of this culture, more research into other fig-infecting viruses is required, as well as the establishment of a programme for the sanitary and clonal selection of local fig varieties to be used as healthy mother plants for multiplication and distribution to producers.

## Figures and Tables

**Table 1:** Primer sets used in RT-PCR for the identification of seven fig viruses.

Virus	Primer (5′–3′)	Amplified product (bp)	Amplified region	References
FMV	E5-s CGGTAGCAAATGGAATGAAA E5-a AACACTGTTTTTGCGATTGG	302	RdRp	Elbeaino et al. [31]

FLMaV-1	N17-s CGTGGCTGATGCAAAGTTTA N17-a GTTAACGCATGCTTCCATGA	352	HSP70	Elbeaino et al. [23]

FLMaV-2	F3-s GAACAGTGCCTATCAGTTTGATTTG F3-a TCCCACCTCCTGCGAAGCTAGAGAA	360	HSP70	Elbeaino et al. [32]

FMMaV	LM3-s AAGGGGAATCTACAAGGGTCG LM3-a TATTACGCGCTTGAGGATTGC	311	HSP70	Elbeaino et al. [25]

FCV	R1-s TCGATTGTCTTTGGAGAGG R1-a CGCATCCACAGTATCCCATT	353	RdRp	Elbeaino et al. [24]

FFkaV	d8-s ATGACGACTGTCAACTCCCT d8-a TTAAGCCAGGGTGGGAGTGTTG	270	RdRp	Elbeaino et al. [20]

FLV-1	CPtr1-s CCATCTTCACCACACAAATGTC CPtr2-a CAATCTTCTTGGCCTCCATAAG	389	CP	Gattoni et al. [33]

**Table 2:** Incidence of FMV, FLMaV-1, FLMaV-2, FMMaV, FFkaV, FLV-1, and FCV infections in samples from three Egyptian governorates and distinct fig varieties using RT-PCR assays.

Tested viruses																		
Governorate	Variety	Tested trees	Infected trees		FMV		FLMaV-1		FLMaV-2		FMMaV		FFkaV		FLV-1		FCV	
		No.	NO.	%	NO.	%	NO.	%	NO.	%	NO.	%	NO.	%	NO.	%	NO.	%
Mersa Matruh	Sultany	60	42	70	36	60	7	11.6	13	21.6	—	00	—	00	—	00	—	00
	Kommathri	15	10	66.6	8	53.3	2	13.3	4	26.6	—	00	—	00	—	00	—	00
	El-Adasy	10	6	60	4	40	1	10	2	20	—	00	—	00	—	00	—	00
	Unknown varieties	15	10	66.6	9	60	2	13.3	3	20	—	00	—	00	—	00	—	00
	Total	100	68	68	57	57	12	12	22	22	—	00	—	00	—	00	—	00

Ismailia	Sultany	40	25	62.5	18	45	4	10	9	22.5	—	00	—	00	—	00	—	00
	Kommathri	10	5	50	5	40	1	10	2	20	—	00	—	00	—	00	—	00
	Unknown varieties	20	12	60	9	45	2	10	5	25	—	00	—	00	—	00	—	00
	Total	70	42	60	32	45.7	7	10	16	22.8	—	00	—	00	—	00	—	00

Giza	Sultany	35	20	57.1	14	40	3	8.5	7	20	—	00	—	00	—	00	—	00
	Unknown varieties	15	7	46.6	5	33.3	2	13.3	3	20	—	00	—	00	—	00	—	00
	Total	50	27	54	19	38	5	10	10	20	—	00	—	00	—	00	—	00
Total		220	137		108		24		48									
Mean infection rate %				62.27		49		10.9		21.8		00		00		00		00

## Data Availability

The outcome of this preliminary work extends the knowledge on the spread of fig viruses in the Mediterranean region, particularly in Egypt for which a little information was previously available. This is the report of FLMaV-1, FLMaV-2, and FMV occurring in the Egyptian fig orchards. Although this assessment was limited to 220 trees, the results obtained clearly indicate how the sanitary status of fig crop has deteriorated in Egypt (62.27% of viral infection). Particularly, worrying is the incidence of FMV, since this has proved to be the unique virus closely correlated with the FMD. High incidence of FMV is not surprising considering the way this virus spreads in figs through infected propagating material (cuttings and grafting), and natural vectors (eriophyid mites). In Egypt, there is no information on the presence of *Aceria ficus* (Eriophyidae) and *Planococcus ficus* (Pseudococcidae), the recognized vectors of FMV and FLMaV-2, respectively. However, such presence in the Egyptian orchards would likely aggravate the sanitary status and the level of infections in the surveyed areas. The several FMV-infected samples found in association with most of the mosaic symptoms in the field further confirms what was previously reported regarding the aetiology of FMV. Nevertheless, this postulate was infringed by a few cases where FMV was detected in symptomless fig trees. Whether this is due to the virus strain present in the country or to the biological response of some Egyptian fig varieties to FMV infection remains to be determined. The knowledge we have gained on the incidence of virus diseases of fig in Egypt provides information on which sanitary selection, sanitation, and certification programmes can be initiated for the production of healthy propagating plant material of fig in this country.

## Supplementary Materials

Supplementary Materials Multiple sequence alignments of Egyptian isolates for fig mosaic virus (FMV), fig leaf mottle-associated virus-1 (FLMaV-1), and fig leaf mottle-associated virus-2 (FLMaV-2) comparing with the isolates available in GenBank.
