# Supplementary material for: Incidence, Molecular Detection, and Partial Nucleotide Sequencing of Some Viruses Causing Fig Mosaic Disease (FMD) on Fig Plants in Egypt
Source: Int J Microbiol. 2022 May 31;2022:2093655. doi: 10.1155/2022/2093655 (PMC9173903; doi:10.1155/2022/2093655)
Supplement: Supplementary Materials — Multiple sequence alignments of Egyptian isolates for fig mosaic virus (FMV), fig leaf mottle-associated virus-1 (FLMaV-1), and fig leaf mottle-associated virus-2 (FLMaV-2) comparing with the isolates available in GenBank. [file 2093655.f1.docx]

**Supplementary files**


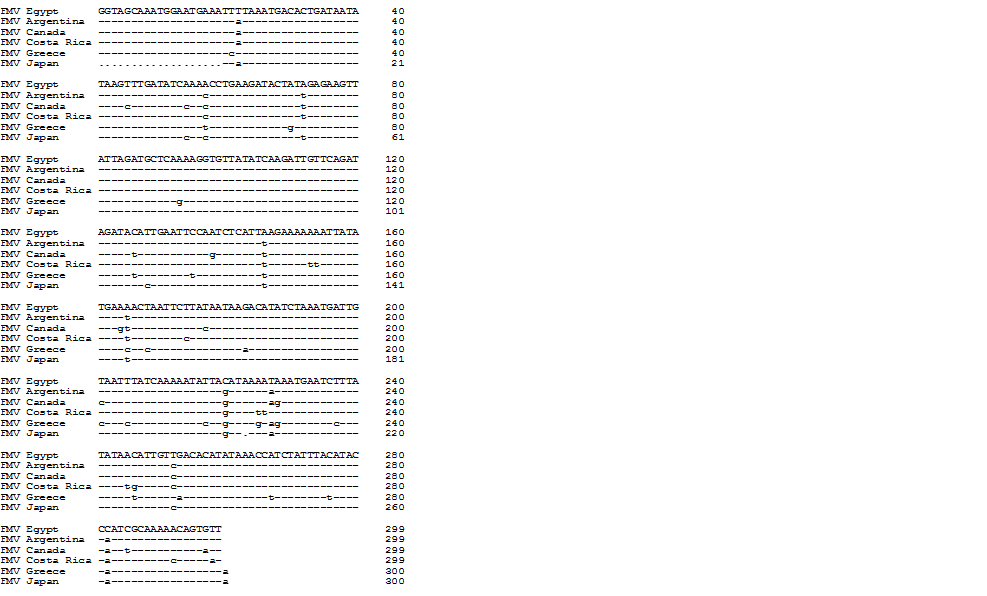


FIGURE: Multiple sequence alignment of the RdRp gene for RNA- dependent RNA polymerase for FMV Egyptian isolate compared with Argentina, Canada, Costa Rica, Greece and Japan isolates

FIGURE: Multiple sequence alignment of the HSP70 gene for RNA- dependent RNA polymerase for FLMaV-1 Egyptian isolate compared with Italy Montenegro, Saudi Arabia, Tunisia and Turkey isolates.

FIGURE: Multiple sequence alignment of the HSP70 gene for RNA- dependent RNA polymerase for FLMaV-2 Egyptian isolate compared with Algeria, Italy, Saudi Arabia, Syria and Tunisia isolates.
